# Impact of baseline cardiovascular risk on the outcomes of intensive blood pressure intervention: a post hoc analysis of the China rural hypertension control project

**DOI:** 10.1186/s12916-024-03494-w

**Published:** 2024-06-20

**Authors:** Guozhe Sun, Chang Wang, Ning Ye, Chuning Shi, Nanxiang Ouyang, Lixia Qiao, Guangxiao Li, Linlin Zhang, Yao Yu, Zhi Li, Ying Zhou, Zihan Chen, Shu Zhang, Pengyu Zhang, Danxi Geng, Wei Miao, Songyue Liu, Yingxian Sun

**Affiliations:** Department of Cardiology, The First Hospital of China Medical University, 155 Nanjing North Street, Heping District, Shenyang, 110001 China

**Keywords:** Baseline cardiovascular risk, Cardiovascular events, Intensive blood pressure lowering strategy

## Abstract

**Background:**

The 2018/2023 ESC/ESH Guidelines underlined a gap how baseline cardiovascular disease (CVD) risk predicted blood pressure (BP) lowering benefits. Further, 2017 ACC/AHA Guideline and 2021 WHO Guideline recommended implementation studies about intensive BP control. Now, to bridge these guideline gaps, we conducted a post hoc analysis to validate whether the baseline CVD risk influences the effectiveness of the intensive BP control strategy, which was designed by China Rural Hypertension Control Project (CRHCP).

**Methods:**

This is a post hoc analysis of CRHCP, among which participants were enrolled except those having CVD history, over 80 years old, or missing data. Subjects were stratified into quartiles by baseline estimated CVD risk and then grouped into intervention and usual care group according to original assignment in CRHCP. Participants in the intervention group received an integrated, multi-faceted treatment strategy, executed by trained non-physician community health-care providers, aiming to achieve a BP target of < 130/80 mmHg. Cox proportional-hazards models were used to estimate the hazard ratios of outcomes for intervention in each quartile, while interaction effect between intervention and estimated CVD risk quartiles was additionally assessed. The primary outcome comprised myocardial infarction, stroke, hospitalization for heart failure, or CVD deaths.

**Results:**

Significant lower rates of primary outcomes for intervention group compared with usual care for each estimated CVD risk quartile were reported. The hazard ratios (95% confidence interval) in the four quartiles (from Q1 to Q4) were 0.59 (0.40, 0.87), 0.54 (0.40, 0.72), 0.72 (0.57, 0.91) and 0.65 (0.53, 0.80), respectively (all *P*s < 0.01). There’s no significant difference of hazard ratios by intervention across risk quartiles (*P* for interaction = 0.370). Only the relative risk of hypotension, not symptomatic hypotension, was elevated in the intervention group among upper three quartiles.

**Conclusions:**

Intensive BP lowering strategy designed by CRHCP group was effective and safe in preventing cardiovascular events independent of baseline CVD risk.

**Trial registration:**

The trial is registered with ClinicalTrials.gov, NCT03527719.

## Background

High blood pressure (BP) is the most important risk factor of cardiovascular disease (CVD), supported by robust evidence [1–3]. Recent studies suggested that incorporating CVD risk as well as BP levels into consideration had been proved to be more advantageous in guiding antihypertensive treatment to reduce adverse events [4–6]. Accordingly, the 2021 WHO Guideline set the goal for hypertensive patients with high CVD risk as systolic BP (SBP) < 130 mmHg [7]. The 2017 ACC/AHA Guideline advocated for a BP target of < 130/80 mmHg in hypertensive adults with either known CVD or a 10-year ASCVD risk of 10% or higher [8]. Therefore, all these guidelines have taken baseline CVD risk into account when setting a BP target, recommended intensive BP in high baseline CVD risk population, but not covered low to moderate risk population. Further, the 2018 ESC/ESH Guideline directly pointed out “What baseline level of CVD risk predicts treatment benefit?” in the section “Gaps in the evidence” [9]. Meanwhile, the 2023 ESH Guideline underlined the gap “BP thresholds and targets in low to moderate risk individuals” [10].

In addressing the critical question of optimal BP management, it is imperative to not only consider the efficacy of BP lowering strategies in reducing major CVD events and all-cause mortality, but also to rigorously evaluate the benefits in relation to potential harms. This balance assessment is especially vital in the context of intensive BP lowering strategies, where the margin between therapeutic gain and adverse effects can be narrow [11]. In Systolic Pressure Intervention Trial (SPRINT) study, patients with higher baseline estimated CVD risk without diabetes and stroke, were more likely to benefit from intensive treatment [12]. However, only 25% US adults with elevated SBP and high estimated CVD risk would have qualified for SPRINT, showing that intensive BP treatment recommendation should not be extended until more definitive evidence [13]. The Action to Control Cardiovascular Risk in Diabetes (ACCORD) trial demonstrated that in patients with type 2 diabetes and high CVD risk, intensive treatment (SBP < 120 mmHg) resulted in a lower occurrence of the primary outcome compared to standard treatment even though this difference did not reach statistical significance [14]. The coverage of the population in these two randomized clinical trials was partial. And the effect of intensive BP control in the general population with hypertension at low to moderate risk remains indeterminate. Thus, further studies on the intensive BP lowering strategies among all baseline estimated CVD risk stratification are necessary.

As the 2017 ACC/AHA Guideline recommended in the evidence gaps, implementation studies that demonstrated the practicality for intensive BP lowering interventions were needed [8]. And the 2021 WHO Guideline also highlighted research gaps exploring the implementation of CVD risk-based antihypertensive treatment in primary health care settings [7]. Our China Rural Hypertension Control Project (CRHCP) trial's outcomes highlight the effectiveness and safety of an intensive BP strategy (< 130/80 mmHg) led by non-physician community health-care providers [15]. Unlike prior investigations focused on intensive BP management, our research integrated general hypertensive patients, systematically including all baseline CVD risk strata. The aim of this study is to address the existing guideline gap by conducting a post hoc analysis of the CRHCP trial, with the objective of elucidating the scientific inquiry into the variability of intensive BP effects across different risk stratification. Based on the data of this implementation study, we hypothesized that grouping hypertensive patients by baseline CVD risk estimation would identify optimal use of this intensive BP lowering strategy.

## Methods

### Data and Ethics

This study was a post hoc analysis of the CRHCP trial, which was designed to test the effectiveness of a non-physician community health-care provider-led intensive BP intervention (< 130/80 mmHg) compared with usual care among hypertensive patients. It was approved by the ethics committees of the First Hospital of China Medical University and performed in 326 villages from three provinces (Liaoning, Shanxi and Hubei) in rural China. All participants have signed informed consent at screening visits. All data used in this study were obtained from the CRHCP.

### Study Design for CRHCP

The design and main results of CRHCP have been published [15]. In a word, as an open-label, blinded-endpoint, cluster-randomized trial, participants aged 40 years or older, with an untreated SBP ≥ 140 mmHg or a diastolic BP (DBP) ≥ 90 mmHg (≥ 130 mmHg and ≥ 80 mmHg for those with high CVD risk or if currently taking antihypertensive medication) were recruited. 326 villages were assigned to a non-physician community health-care provider-led intervention or usual care randomly. In the intervention group, providers were trained and implied antihypertensive management to achieve a BP goal of < 130/80 mmHg. A total of 33,995 participants were enrolled from May 8 to November 28, 2018 and followed for clinical events over 36 months. More details on the outcomes and procedure could be available in published papers [16].

### Randomization and masking

**Fig. 1:**
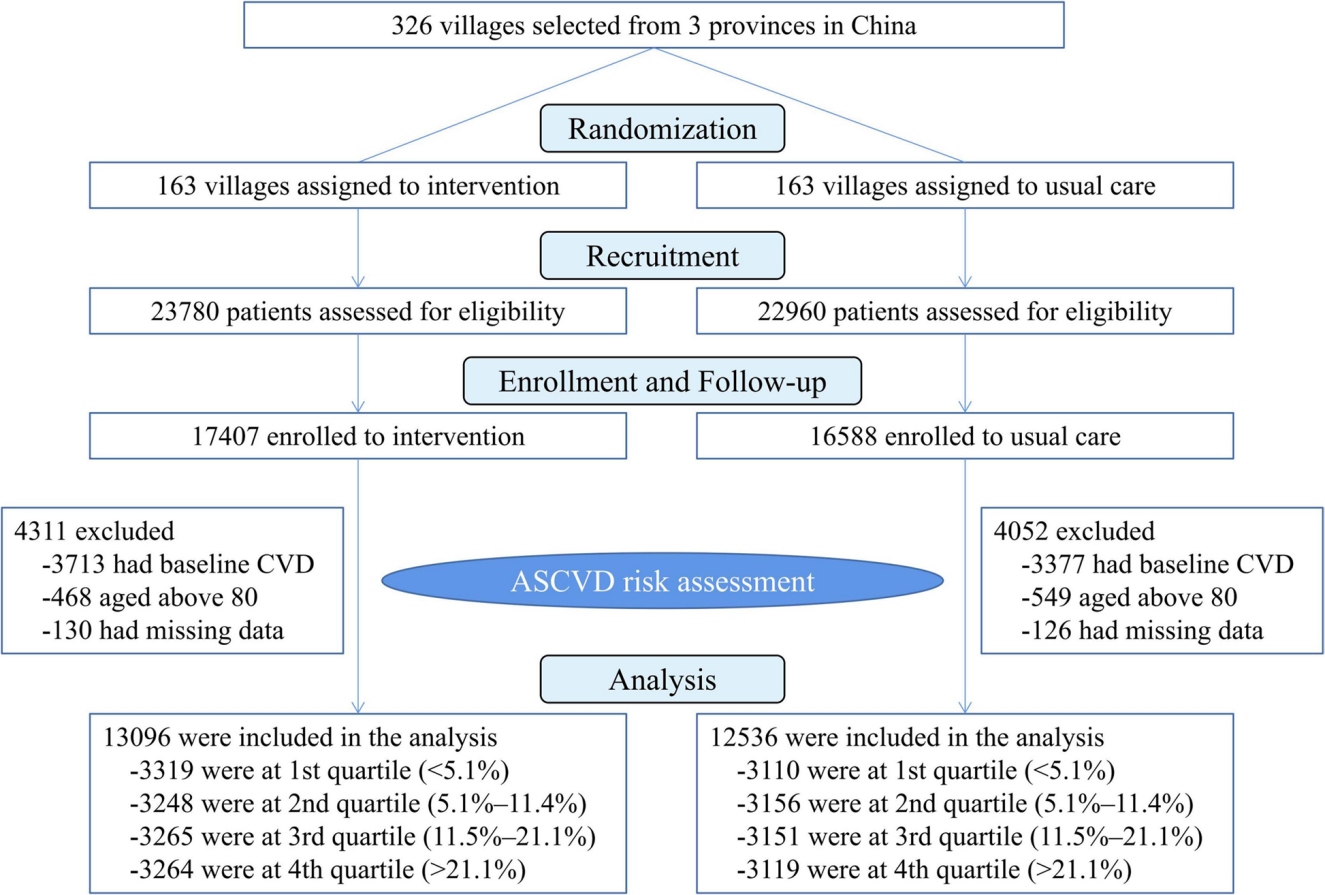
Flowchart of the study. The randomization, recruitment, enrollment and baseline CVD risk quartile of this study are shown

Randomization and masking for this study was mainly located at CRHCP. Randomization was stratified by provinces, counties, and townships. A total of enrolled 163 villages were randomly assigned to intervention and 163 villages to usual care by a biostatistician from the Tulane University Translational Science Institute (Fig. 1). Since it’s a cluster-based implementation program, the participants, providers, and research staff for data collection were unblinded. However, the randomization assignments were concealed before the completion of recruitment and enrollment. Besides, the process of clinical outcome assessment was blinded to randomization.

### Participants stratifications and Groups for this study

This current study screened all eligible participants from CRHCP, among which participants who had a history of CVD, over 80 years old, or missing data were excluded from this analysis since the calculation of ASCVD risk by the American College of Cardiology/American Heart Association Pooled Cohort Equations. We used the same estimation method as outlined in our main article [15] and the Pooled Cohort Risk Equations were accepted and recognized worldwide. During stratification, the distribution of CVD risk was first assessed and it was found that the estimated 10-year risk in the lowest one was less than 5.1% if using quartiles, which matched the low risk category (less than 5%) in risk assessment system. Thus, the whole enrolled subjects were stratified into quartiles by baseline 10-year CVD risk estimation. For each quartile, subjects were assigned to intervention or usual care group according to the original randomization of CRHCP. The flowchart of this study was presented in Fig. 1.

### Intervention and Measurements

Demographic data was collected at baseline, while laboratory indexes were measured at both baseline and 36 months. Biological sex was self-reported by participants. BP measurements were taken three times every six months, with participants resting in a seated position for five minutes each time. The collected measurements were promptly submitted to the study data center, where they were pooled and analyzed to identify trends in BP control.

To ensure a comprehensive overview of health outcomes, cardiovascular incidents, and other potential adverse events were systematically monitored by the CRHCP study group every six months. Detailed information about cardiovascular conditions and mortality rates was amassed through a standardized questionnaire, which included an extensive array of variables such as medical history, CVD risk factors, and specific symptoms. The study also rigorously tracked a range of conditions including injurious falls, all forms of hypotension, and syncope, while electrolyte levels and renal function, indicated by estimated glomerular filtration rate (eGFR), were documented as integral components of the adverse outcomes assessment.

Participants in the intervention group were given the same management across all estimated CVD risk stratification. An integrated, multi-faceted treatment strategy was executed by trained non-physician community health-care providers, aiming to achieve a BP target of < 130/80 mmHg. In contrast, participants in the control group were subject to standard care practices. The community health-care providers in the intervention group, under the supervision of hypertension specialists and primary care physicians, were rigorously trained in a comprehensive, protocol-based antihypertensive regimen. This regimen included in-depth instruction on treatment algorithms, pharmacological selection, contraindications, and titration strategies. Furthermore, these providers received extensive training in patient health education, covering essential aspects such as home BP monitoring, adherence to medication, and lifestyle modifications. The role of these community health providers was multifaceted, encompassing the initiation and adjustment of antihypertensive medications, direct medication delivery to patients, health coaching, instruction in home BP monitoring practices, and the organization of social support groups. They received a portion of their salaries and performance-based incentives from research grants for study-related activities. To promote engagement and adherence, patients in the intervention group were provided with monthly supplies of antihypertensive medications at discounted rates or free of charge, accompanied by complimentary home BP monitoring devices. Additionally, they received consistent health coaching sessions facilitated by a dedicated team of non-physician community healthcare providers, ensuring a comprehensive, supportive, and well-monitored treatment environment.

### Study outcomes

We examined the primary outcomes in this implementation study which was defined as the first occurrence of major CVD events composing of myocardial infarction, stroke, heart failure requiring hospitalization or CVD deaths during the 36-month follow-up. We also examined adverse events to reflect intervention safety. Serious adverse events included deaths and hospitalizations in this analysis. Besides, injurious falls, hypotension, symptomatic hypotension, syncope, electrolyte abnormalities, and renal outcomes were compared and presented. Details of events adjudication had been published in the previous article by our study team [15, 16]. All study outcomes were adjudicated by the Endpoint Adjudication Committee.

### Statistical analysis

The predicted ASCVD risk was calculated based on the American College of Cardiology/American Heart Association Pooled Cohort Equations [17]. The eGFR was calculated based on the 2021 Chronic Kidney Disease Epidemiology Collaboration Creatinine Equation [18]. Baseline demographics, risk factors, and end point BP of intervention and usual care treatment stratified by estimated CVD risk were calculated as mean ± standard deviation for continuous variables and number (percentage) for categorical variables. They were compared with the use of the Chi-square test, Wilcoxon rank-sum test, and the Student’s t-test, as appropriate. Tests for trend across quartiles of estimated CVD risk were conducted by modeling the quartiles as a continuous variable in linear regression models for continuous variables and the Cochran-Armitage test for trend for categorical variables. The Kaplan–Meier curves were drawn and log-rank test was used to detect the difference of event incidences between groups. Mixed-effect Cox proportional-hazards models were used to estimate the hazard ratios (HRs) and 95% confidence intervals (CIs) of primary outcomes, all-cause mortality and CVD mortality associated with the intervention in each quartile, setting the village as a random effect. Baseline covariates including age, sex, cigarette smoking, use of antihypertensive medication, and baseline SBP, low-density lipoprotein cholesterol, and fasting plasma glucose were also adjusted. To test the interaction effect, a multiplicative interaction term between intervention and estimated CVD risk quartiles was additionally introduced to the regression model. We also calculated the absolute risk reduction (ARR) and number of person-years needed to treat (NNT). In our study, we employed the “iri” statement within the Stata software to compute ARR. ARR is defined as the absolute disparity in risk between two groups, specifically the usual care group and the intervention group. The ARR was calculated using the formula: ARR = Risk in the usual care group—Risk in the intervention group. It's important to note that the risks were expressed as rates per person-year. Benjamini–Hochberg method was applied to control the false discovery rate (FDR) in the subgroup analysis for the comparison of primary outcome events, all-cause death, and CVD death. Two-tailed *P* values < 0.05 were considered statistically significant for all analyses. Statistical analysis was conducted with the use of Stata MP 17.0 and R software 4.2.0.

## Results

### Participants

This current study screened all eligible 33,995 participants from CRHCP. Among which, 7090 participants with baseline CVD, 1018 of aged over 80 years, and 256 with incomplete data were excluded from this study, leaving a total of 25,632 subjects (75.4% of CRHCP participants) in the final analysis. Among these patients, 13,096 were assigned to intensive BP management and 12,536 were assigned to usual care according to the original randomization. The first quartile of the enrolled participants had a 10-year CVD risk of < 5.1%, the second quartile of the participants had a risk of 5.1%-11.4%, the third and fourth quartile was 11.5%-21.1% and > 21.1%, respectively (Fig. 1). Baseline characteristics between two groups across estimated CVD risk quartiles were presented and compared (Table 1). There was a significant trend of increasing age, mean baseline level of SBP, total cholesterol (TC) and low density lipoprotein-cholesterol (LDL-C), proportion of smoking and drinking, and prevalence of diabetes with increasing quartile. And lower estimated CVD risk subgroup had more female participants and higher mean high-density lipoprotein-cholesterol (HDL-C). These trends were reasonable since most of variables were the component of the 10-year CVD risk algorithm. There were small numerical value differences of mean levels of age, SBP, uric acid and eGFR and proportions of female, education, smoking and drinking between the intervention and usual care group at some quartiles although there were statistically significant. However, the mean plasma glucose, TC, and LDL-C were similar between two groups at each estimated CVD risk quartile. Remarkably, the mean 10-year CVD risk was balanced across the intervention and usual care group at all estimated CVD risk quartiles except the fourth.

**Table 1:** Baseline characteristics of the participants by quartile of estimated 10-year ASCVD risk and groups

Characteristics	1st quartile (< 5.1%)		2nd quartile (5.1%–11.4%)		3rd quartile (11.5%–21.1%)		4th quartile (> 21.1%)	
	**Intervention**	**Usual care**	**Intervention**	**Usual care**	**Intervention**	**Usual care**	**Intervention**	**Usual care**
Number of participants	3319	3110	3248	3156	3265	3151	3264	3119
Mean age (SD), years	52.1 (5.2)	52.5 (5.1)	59.7 (5.7)	60.1 (5.7)	64.2 (5.8)	64.7 (5.7)	70.6 (5.6)	70.5 (5.7)
Female sex, n (%)	2971 (89.5)	2847 (91.5)	2410 (74.2)	2357 (74.7)	1674 (51.3)	1645 (52.2)	1213(37.2)	1148 (36.8)
Education, n (%)								
Primary school or less	1748 (52.7)	1798 (57.9)	2104 (64.9)	2095 (66.5)	2181 (66.9)	2190 (69.6)	2333 (71.6)	2186 (70.2)
Junior high school	1318 (39.8)	1117 (36.0)	921 (28.4)	837 (26.6)	848 (26.0)	763 (24.3)	760 (23.3)	779 (25.0)
High school	199 (6.0)	165 (5.3)	190 (5.9)	196 (6.2)	208 (6.4)	175 (5.6)	154 (4.7)	128 (4.1)
College or higher	50 (1.5)	27 (0.9)	25 (0.8)	22 (0.7)	23 (0.7)	17 (0.5)	13 (0.4)	19 (0.6)
Cigarette smoking, n (%)								
Never smoked	3139 (94.6)	2979 (95.8)	2621 (80.7)	2528 (80.1)	1976 (60.5)	1893 (60.1)	1667 (51.1)	1484 (47.6)
Former smokers	94 (2.8)	60 (1.9)	165 (5.1)	185 (5.9)	286 (8.8)	305 (9.7)	351 (10.8)	326 (10.5)
Current smokers	86 (2.6)	71 (2.3)	462 (14.2)	443 (14.0)	1003 (30.7)	953 (30.2)	1246 (38.2)	1309 (42.0)
Weekly alcohol drinking, n (%)	243 (7.3)	193 (6.2)	485 (14.9)	462 (14.6)	830 (25.4)	776 (24.7)	745 (22.9)	811 (26.0)
Duration of hypertension	6 (4–8)	6 (3–8)	7 (4–10)	7 (4–10)	8 (5–12)	7 (4–11)	8 (5–13)	8 (5–12)
Median (IQR), years								
Use of antihypertensive medications, n (%)	1864 (56.2)	1509 (48.5)	1892 (58.3)	1626 (51.5)	1862 (57.0)	1624 (51.5)	2058 (63.1)	1773 (56.8)
History of diabetes, n (%)	76 (2.3)	82 (2.6)	215 (6.6)	184 (5.8)	271 (8.3)	236 (7.5)	511 (15.7)	478 (15.3)
History of chronic kidney disease, n (%)	15 (0.5)	14 (0.5)	17 (0.5)	15 (0.5)	14 (0.4)	16 (0.5)	22 (0.7)	12 (0.4)
Mean body mass index (SD), kg/m^2^	27.0 (3.8)	26.9 (3.8)	26.5 (3.8)	26.3 (3.8)	25.9 (3.8)	25.6 (3.6)	25.3 (3.8)	25.2 (3.8)
Mean systolic blood pressure (SD), mmHg	151.1 (14.5)	149.2 (13.5)	154.5 (16.3)	153.2 (15.6)	156.8 (16.9)	156.1 (16.5)	164.1 (19.4)	162.6 (18.8)
Mean diastolic blood pressure (SD), mmHg	90.4 (9.3)	89.6 (9.1)	88.4 (10.4)	87.9 (10.1)	87.6 (10.9)	87.2 (10.7)	86.9 (11.3)	86.3 (11.2)
Mean total cholesterol (SD), mg/dL	188.9 (35.9)	189.2 (35.2)	198.3 (37.8)	197.5 (37.9)	197.8 (39.6)	196.6 (39.7)	197.2 (39.9)	197.7 (40.5)
Mean low-density lipoprotein cholesterol (SD), mg/dL	99.2 (28.5)	99.5 (28.3)	105.3 (31.2)	105.6 (30.6)	106.7 (32.5)	105.8 (31.9)	107.9 (32.6)	108.0 (33.4)
Mean high-density lipoprotein cholesterol (SD), mg/dL	57.6 (12.8)	57.2 (12.3)	56.6 (13.5)	56.3 (12.9)	56.3 (13.9)	55.8 (14.0)	54.6 (13.8)	54.5 (13.9)
Mean plasma glucose (SD), mg/dL	104.8 (31.8)	104.9 (28.9)	111.2 (34.2)	110.1 (34.8)	111.0 (35.4)	111.8 (37.6)	116.6 (42.6)	115.0 (39.1)
Mean uric acid (SD), mg/dL	4.7 (1.5)	4.6 (1.3)	5.0 (1.4)	5.0 (1.4)	5.2 (1.5)	5.2 (1.5)	5.3 (1.4)	5.4 (1.5)
Mean estimated glomerular filtration rate (SD), mL/min/1.73 m^2a^	104.5 (10.0)	104.0 (9.8)	98.8 (10.3)	98.2 (10.5)	95.5 (11.1)	95.1 (10.8)	89.8 (12.3)	89.7 (12.0)
Mean 10-year risk for atherosclerotic cardiovascular disease (SD), %^b^	2.8 (1.2)	2.8 (1.2)	8.0 (1.9)	8.0 (1.8)	15.9 (2.8)	15.9 (2.8)	31.6 (9.1)	31.0 (8.6)

*GFR* glomerular filtration rate, *IQR* interquartile range, *SD* standard deviation

^a^Estimated glomerular filtration rate was calculated based on the 2021 Chronic Kidney Disease Epidemiology Collaboration creatinine equations

^b^Atherosclerotic cardiovascular disease risk was calculated based on the American College of Cardiology and American Heart Association Pooled Cohort Equations

### Blood pressure at last follow-up

The achieved BP at the end of follow-up was summarized in Table 2 according to quartiles of 10-year CVD risk and CRHCP treatment arm. Besides, the mean BP levels were added and compared. As a result, the mean SBP of the intervention group decreased to 126.0 mmHg while the mean SBP of the usual care group was 147.3 mmHg. For each quartile, the reductions in SBP by intervention were all significant (all *P*s < 0.001). The four groups with the intensive intervention had similar level of SBP, but in the usual care group there was still a significant trend of increasing mean SBP with ascending quartile (*P* for interaction < 0.001).

**Table 2:** Systolic and diastolic blood pressure at the last follow-up stratified by baseline estimated CVD risk quartile

Variables	Mean (95%CI)		Net difference (95% CI)^a^	*P* value	*P* value for interaction
	**Intervention**	**Usual care**			
Achieved SBP					
Q1	124.7 (124.2, 125.2)	142.5 (141.6, 143.4)	-17.8 (-18.9, -16.8)	< 0.001	< 0.001
Q2	125.4 (124.8, 125.9)	145.2 (144.3, 146.0)	-19.8 (-20.8, -18.8)	< 0.001	
Q3	126.6 (126.1, 127.2)	149.4 (148.6, 150.3)	-22.8 (-23.8, -21.8)	< 0.001	
Q4	127.5 (126.9, 128.2)	152.7 (151.7, 153.7)	-25.2 (-26.4, -24.0)	< 0.001	
Achieved DBP					
Q1	74.7 (74.3, 75.0)	84.2 (83.6, 84.7)	-9.48 (-10.1, -8.8)	< 0.001	0.460
Q2	73.0 (72.7, 73.4)	82.4 (81.9, 82.9)	-9.4 (-10.0, -8.8)	< 0.001	
Q3	72.9 (72.5, 73.3)	82.4 (81.8, 82.9)	-9.5 (-10.1, -8.8)	< 0.001	
Q4	72.0 (71.6, 72.4)	81.1 (80.5, 81.7)	-9.1 (-9.8, -8.4)	< 0.001	

^a^ The means were adjusted for cluster effects, age, and sex using generalized linear models

The mean achieved DBP of the intervention group reached to 73.2 mmHg while it was 82.5 mmHg in the usual care group. Similar with SBP, the reductions by intervention were all significant for each quartile (all *P*s < 0.001). The BP levels across quartiles between intervention and usual care group had similar trends (*P* for interaction = 0.959).

### Primary outcomes and mortality

During a median of 2.95 ± 0.33 years, the events of primary outcomes were 965 in total (330 from intervention group and 635 from usual care group) (Table 3). The HRs of primary outcomes with intervention in the first, second, third, and fourth estimated CVD risk quartile were 0.59 (95% CI: 0.40–0.87), 0.54 (95% CI: 0.40–0.72), 0.72 (95% CI: 0.57–0.91), and 0.65 (95% CI: 0.53–0.80), respectively (all *P*s < 0.01). No significant difference of HR by intervention across quartiles was detected (*P* for interaction = 0.370). Similar results were reported after adjustment for baseline age, sex, smoking, use of antihypertensive medication, SBP, LDL-C, and fasting plasma glucose. The ARR of primary outcomes increased gradually from the first to fourth quartile in general, with the highest ARR of 1.05 (95% CI: 0.58–1.52) in the fourth quartile. And the number of NNT for the primary outcome gradually declined from the first to fourth quartile in general.

**Table 3:** Hazard ratios of intervention for primary outcome events, all-cause death and cardiovascular death by estimated 10-year ASCVD risk quartile

Study outcomes	Intervention		Usual care		Hazard ratio	*P* value	Adjusted hazard ratio	*P* value	*P* value for interaction	Absolute Risk	NNT
	**No. of events**	**Rate, % per year**	**No. of events**	**Rate, % per year**	(95% CI)^a^		(95% CI)^a^^b^			Reduction (95% CI)	
The primary outcome of cardiovascular disease (myocardial infarction, stroke, heart failure, or cardiovascular disease death)											
1st Quartile (< 5.1%)	41	0.42	65	0.71	0.59 (0.40, 0.87)	0.008	0.53 (0.36, 0.79)	0.002	0.370	0.29 (0.08 to 0.51)	345
2nd Quartile (5.1%–11.4%)	77	0.81	138	1.51	0.54 (0.40, 0.72)	< 0.001	0.52 (0.39, 0.70)	< 0.001		0.70 (0.39 to 1.01)	143
3rd Quartile (11.5%–21.1%)	126	1.33	166	1.84	0.72 (0.57, 0.91)	0.009	0.71 (0.57, 0.90)	0.005		0.50 (0.15 to 0.87)	200
4th Quartile (> 21.1%)	86	2.01	266	3.06	0.65 (0.53, 0.80)	< 0.001	0.63 (0.51, 0.77)	< 0.001		1.05 (0.58 to 1.52)	95
Death from all causes											
1st Quartile (< 5.1%)	24	0.24	36	0.39	0.62 (0.37, 1.04)	0.097	0.61 (0.36, 1.02)	0.120	0.560	0.15 (-0.01 to 0.31)	667
2nd Quartile (5.1%–11.4%)	51	0.53	62	0.67	0.80 (0.55, 1.16)	0.240	0.81 (0.56, 1.18)	0.28		0.14 (-0.09 to 0.36)	714
3rd Quartile (11.5%–21.1%)	80	0.83	104	1.13	0.74 (0.55, 1.00)	0.192	0.76 (0.56, 1.02)	0.091		0.29 (0.01 to 0.58)	345
4th Quartile (> 21.1%)	201	2.13	233	2.59	0.81 (0.66, 1.00)	0.110	0.79 (0.64, 0.97)	0.096		0.46 (0.02 to 0.91)	217
Cardiovascular death											
1st Quartile (< 5.1%)	4	0.04	12	0.13	0.31 (0.10, 0.97)	0.088	0.23 (0.07, 0.73)	0.052	0.067	0.09 (0.01 to 0.17)	1111
2nd Quartile (5.1%–11.4%)	8	0.08	22	0.23	0.35 (0.16, 0.79)	0.048	0.36 (0.16, 0.81)	0.028		0.15 (0.04 to 0.27)	667
3rd Quartile (11.5%–21.1%)	20	0.21	31	0.34	0.61 (0.35,1.08)	0.120	0.63 (0.36, 1.10)	0.100		0.13 (-0.02 to 0.27)	769
4th Quartile (> 21.1%)	53	0.56	69	0.77	0.73 (0.50, 1.06)	0.098	0.67 (0.46, 0.98)	0.052		0.21 (-0.03 to 0.44)	476

*CI* confidence interval, *NNT* number of person-years needed to treat. Confidence intervals were not adjusted for multiple comparisons and should not be used in place of hypothesis testing

^a^ In the mixed-effect Cox proportional-hazards regression models, the village was set as a random effect

^b^ Adjustment for age, sex, smoking, use of antihypertensive medication, baseline systolic blood pressure, low-density lipoprotein cholesterol, and fasting plasma glucose

All-cause and CVD deaths were also compared (Table 3). A total of 356 all-cause deaths occurred among 13,096 participants from the intervention group, while 435 occurred among 12,536 participants from usual care group. And there were 85 and 134 CVD deaths reported in the intervention and usual care group, respectively. The apparent reduction of deaths due to the intervention strategy was observed across each quartile of estimated CVD risk, even though there was no statistical difference of HR for all-cause and CVD mortality between intervention and usual care group (*P*s > 0.05) except for CVD mortality in the second quartile (*P* < 0.05).

**Fig. 2:**
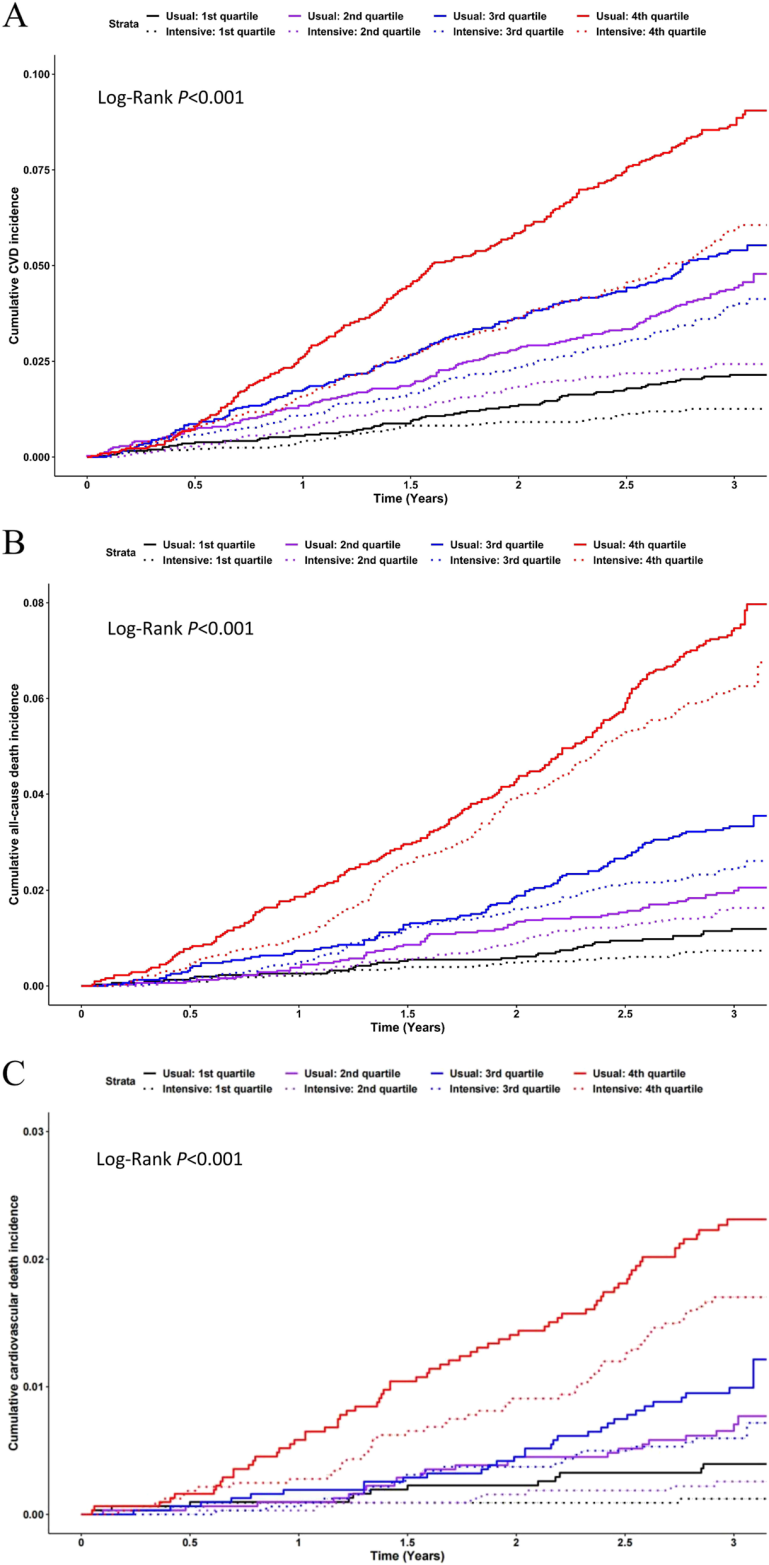
Cumulative incidence of the outcomes for intervention versus usual care group by baseline CVD risk quartile. Cumulative incidence of cardiovascular disease is shown in Figure **A**, cumulative incidence of all-cause mortality is shown in Figure **B**, and cumulative incidence of cardiovascular death is shown in Figure **C**

As shown in Fig. 2, not only the cumulative incidence of primary CVD outcomes but also all-cause death and CVD death increased as the increasing quartile of estimated CVD risk. Obviously, the cumulative incidence curve of CVD events at the fourth quartile under usual care was far away from the other curves. Further, the curve of intervention group was lower than usual care group in each quartile.

### Adverse Events

**Fig. 3:**
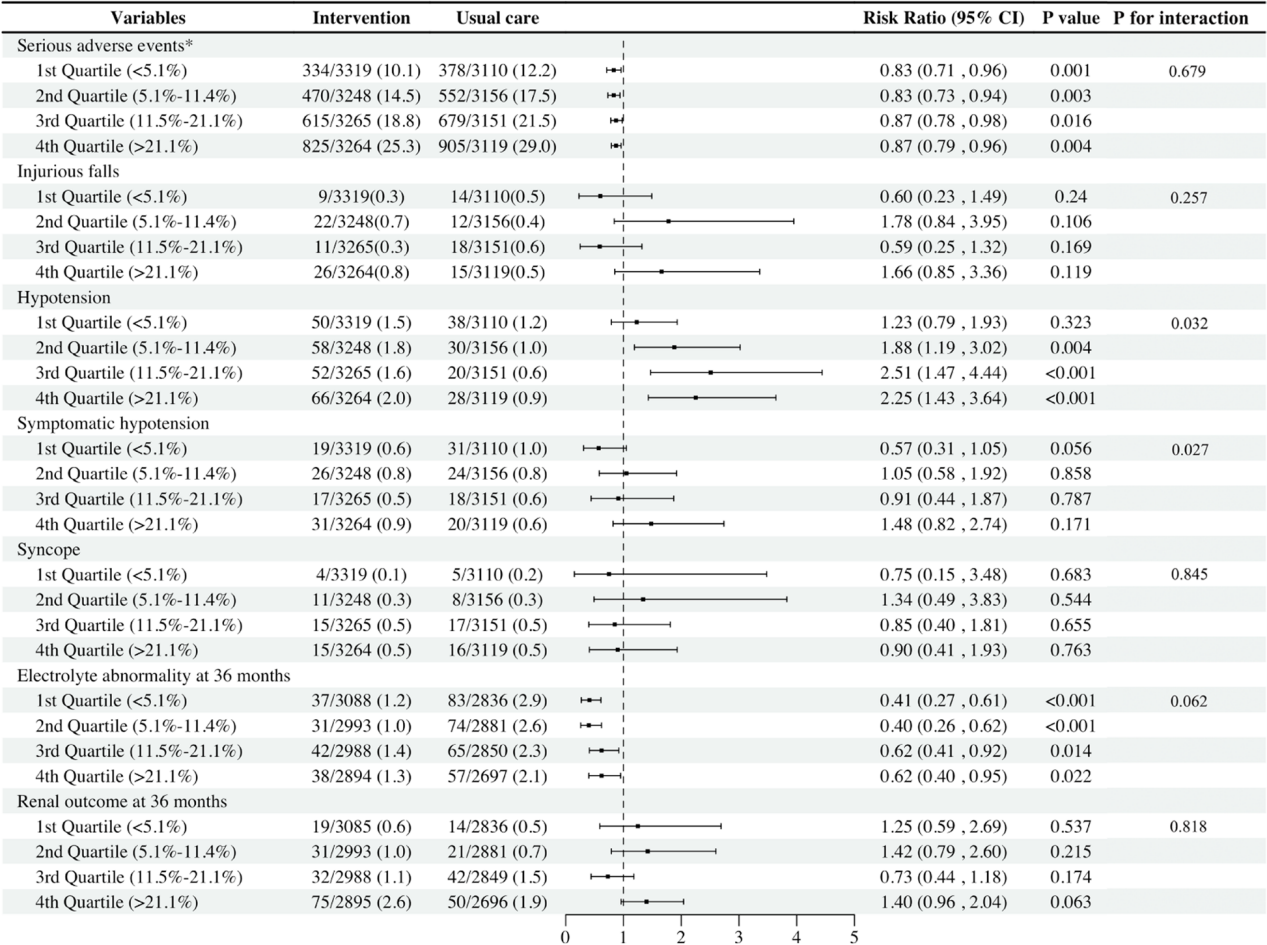
Risk ratios of intervention for adverse events by 10-year ASCVD risk quartile. CI = confidence interval, GFR = glomerular filtration rate. * Serious adverse events included deaths and hospitalizations in this analysis. † Electrolyte abnormality at 36 months means serum sodium < 130 or > 150 mmol/L, or serum potassium < 3.0 or > 5.5 mmol/L. ‡ Renal outcomes at 36 months refers to ≥ 50% reduction in estimated GFR in patients with chronic kidney disease at baseline, or ≥ 30% reduction in estimated GFR to < 60 ml/min/1.73 m^2^ in patients without chronic kidney disease at baseline

We systemically assessed the safety endpoints stratified by quartiles of estimated CVD risk and intervention arms (Fig. 3). Serious adverse events including deaths and hospitalizations were compared, and significant reduction were reported in the intervention group at each quartile of estimated CVD risk (all *P*s < 0.05). Additionally, there was a significant decrease of electrolyte abnormality in the intervention group no matter what baseline estimated CVD risk (all *P*s < 0.05). Among these adverse events, hypotension was increased in the intervention group among the last three quartiles of estimated CVD risk (*Ps* < 0.05), with a significant interaction *p*-value of 0.032 indicating different impacts of the intervention across risk groups. However, there was no significant difference in symptomatic hypotension within any quartile (all *P*s > 0.05), despite an interaction *P*-value of 0.027, suggesting a trend of differential effects across quartiles. Further, injurious falls, syncope, and renal outcomes were also compared between two groups across quartiles, having no statistically difference (all *P*s > 0.05).

## Discussion

This study tested the effectiveness of intensive BP intervention designed by CRHCP group in different baseline estimated CVD risk groups and demonstrated that participants in the intervention group had significant lower rates of primary outcomes than usual care group with similar HR across each quartile. Further analysis showed that the absolute benefits increased gradually from the first quartile to the fourth quartile in general, with the highest ARR in subjects with high baseline estimated CVD risk. In addition, these benefits by intensive intervention strategy didn’t increase patients’ risk of serious adverse events.

Hypertension significantly increases the risk for the onset and progression of CVD [19]. Previous studies have showed the benefits from intensive BP management [20, 21]. As for the impacts of baseline CVD risk estimation on CVD events reduction, a meta-analysis illustrated that BP lowering provided similar protection in each quartile, and the absolute reductions gradually increased as baseline risk increasing. But it couldn’t demonstrated the role of intensive BP lowering strategy [22]. A post-hoc analysis of SPRINT suggested that intensive treatment (SBP < 120 mmHg) would bring more benefit than harm only in patients with a 10-year CVD risk > 18.2% [12]. Other analyses from SPRINT proved that intensive BP reduction was beneficial for subjects with middle or high CVD risk [23, 24]. In Studio Italiano Sugli Effetti Cardiovascolari del Controllo della Pressione Arteriosa Sistolica (Cardio-Sis) study, intensive BP treatment (< 130 mmHg) improved clinical outcomes to a similar extent in patients with and without established CVD. But it’s unclear if categorized by baseline risk [25]. While our current study provided novel and direct evidence that the reduction of primary outcomes from intensive BP treatment (target of < 130/80 mmHg) were significant for all hypertensive subjects with different baseline estimated CVD risk. This finding can bridge the gap identified by ESH in terms of optimal strategies for BP target values applicable to populations with low to moderate risk.

As for the representativeness of enrolled sample, our current study had enough proportion of low estimated CVD risk population with half ≤ 11.4% whereas the SPRINT was ≤ 18.2% [12]. According to the National Health and Nutrition Examination Survey (NHANES), most of US adults having elevated BP were not eligible for SPRINT and would be in an uncertain therapeutic gray zone [13]. While in our current study, the estimated 10-year risk in the lowest quartile was less than 5.1% which matched the low risk category (less than 5%) in risk assessment system established from China in 2020, indicating that it could represent the low-risk population. Therefore, this must make up for the gap put forward by the 2023 ESH Guideline very well that BP thresholds and targets in low to moderate risk individuals [10]. Besides, as the 2018 ESC/ESH Guideline recommending [9], we recruited younger patients than SPRINT and patients with diabetes. In addition, we had a large sample of 25,632 persons without CVD at baseline to better explore the effect of BP lowering on primary prevention in the progression of hypertension. Although there was the same target or goal of BP control, the achieved systolic BP was highly dependent on the level of BP at baseline. An increasing trend of SBP and a decreasing trend of DBP was found from Q1 to Q4 groups. This may be due to the following reasons. Firstly, the average age gradually increases from the Q1 to the Q4 group. With aging, arterial stiffness and pulse pressure typically increase. Therefore, although the SBP increases from Q1 to Q4, the DBP correspondingly decreases. Additionally, our BP targets are set to an SBP of less than 130 mmHg and a DBP of less than 80 mmHg, with both targets required to be met simultaneously. Patients in the Q4 group had lower baseline DBP, making it easier for them to initially achieve the DBP target of less than 80 mmHg. After reaching this DBP target, further medication adjustments primarily focused on reducing SBP to meet the target of less than 130 mmHg. During this process, although SBP significantly decreased, DBP could further decrease to lower levels.

While emphasizing the significance of intensive BP control, it is imperative to prioritize considerations regarding its safety in the real-world setting. Just as the 2017 ACC/AHA Guideline recommended, implementation studies that demonstrated the practicality of SPRINT-like interventions in resource-constrained settings were needed for practice [8]. Considering the generalization of our BP management strategy, safety endpoints were simultaneously assessed. Only hypotension but not symptomatic hypotension was increased due to intensive BP lowering. Additionally, no increases of injurious falls, syncope, electrolyte abnormality, and renal outcomes were reported at any baseline estimated CVD risk, indicating its safety. The risk for renal outcomes was consistent with the African American Study of Kidney Disease and Hypertension (AASK) Study, demonstrating that lower BP did not change the risk of kidney disease [26]. Therefore, this current study proved that the intensive BP treatment strategy designed by CRHCP could be implemented for all hypertensive patients regardless of baseline estimated CVD risk. As the guidelines emphasized, this findings would help guiding physicians or community health workers in considering intensive BP lowering. Especially, the management of hypertension was poor with low-resource in China and this trial gave strong evidence to support the non-physician community health-care provider in the general population.

There were some limitations in the current study. First, CRHCP was a cluster randomized controlled and implementation study, and this was a post hoc analysis which would be susceptible to slight between-group differences. Second, the BP decreased gradually so that the effect of BP lowering would be delayed and we may not yet have observed sufficient outcome difference. Third, as the risk stratification increased, age, SBP, plasma glucose, LDL-C and other risk factors aggravated. But, these factors between two groups at each risk quartiles were similar even though some have statistical significance. And the major aims were located at the influence of baseline estimated CVD risk but not one specialized factor. Finally, NNT, reflecting the benefit of the treatment, was relatively large. Since the intensive BP control strategy significiantly reduced CVD events, it may necessitate a cost-effectiveness analysis, which we will conduct in the future.

## Conclusions

Intensive BP intervention targeting a BP of less than 130/80 mmHg designed by CRHCP study was effective and feasible in all estimated CVD risk groups. The absolute benefits increased along with baseline estimated CVD risk without increasing harms. Therefore, stratification of baseline estimated CVD risk should not serve as an impediment to the implementation of intensive BP lowering strategy. Additionally, these evidences could provide some reference when developing guidelines, especially in the setting of BP target and strategies implementation.

## Data Availability

Data can be requested from YS after publication of this study. Deidentified participant data, data dictionary, and other specified datasets can be requested. The study protocol, statistical analysis plan, and informed consent form will also be made available on request. Specific requests for data will require the submission of a proposal with a valuable research question as assessed by the study steering committee. A data access agreement should be signed.

## Abbreviations

AASK: African American Study of Kidney Disease and Hypertension
ACCORD: Action to Control Cardiovascular Risk in Diabetes
ARR: Absolute risk reduction
BP: Blood pressure
Cardio-sis: Cardiovascolari del Controllo della Pressione Arteriosa Sistolica
CI: Confidence interval
CRHCP: China Rural Hypertension Control Project
CVD: Cardiovascular disease
DBP: Diastolic blood pressure
eGFR: Estimated glomerular filtration rate
FDR: False discovery rate
GFR: Glomerular filtration rate
HDL-C: High-density lipoprotein-cholesterol
HR: Hazard ratio
IQR: Interquartile range
LDL-C: Low density lipoprotein-cholesterol
NHANES: National Health and Nutrition Examination Survey
NNT: Number of person-years needed to treat
SBP: Systolic blood pressure
SD: Standard deviation
SPRINT: Systolic Pressure Intervention Trial
TC: Total cholesterol
